# A Associação de TWEAK com Calcificação da Artéria Coronária em Pacientes com Doença Renal Crônica

**DOI:** 10.36660/abc.20210599

**Published:** 2022-06-06

**Authors:** Mustafa Adem Tatlisu, Adem Atici, Fatma Betul Ozcan, Mehmet Çelik, Eray Kirac, Omer Faruk Baycan, Mustafa Caliskan

**Affiliations:** 1 Istanbul Medeniyet University Istanbul Turquia Istanbul Medeniyet University, Istanbul – Turquia

**Keywords:** Doenças Cardiovasculares, Insuficiência Renal Crônica, Rigidez Vascular, Aterosclerose, Doença da Artéria Coronariana

## Abstract

**Fundamento:**

O receptor fraco indutor de apoptose semelhante a fator de necrose tumoral solúvel (sTWEAK) é um membro da superfamília de TNF que tem um papel crítico na proliferação e inflamação na circulação arterial.

**Objetivos:**

Este estudo prospectivo tem o objetivo de mostrar a relação entre os níveis de sTWEAK e calcificação da artéria coronária (CAC) em pacientes com doença renal crônica (DRC).

**Métodos:**

Este estudo prospectivo incluiu 139 pacientes consecutivos que passaram por angiografia coronariana por tomografia computadorizada, por qualquer motivo, para síndromes coronarianas agudas, de agosto de 2020 a fevereiro de 2021. Um total de 12 pacientes foi excluído do estudo devido aos critérios de exclusão. Os pacientes foram divididos em dois grupos com base em terem um escore CAC menor que 400 (n=84) ou um escore de 400 ou mais (n=43). A significância foi presumida em p-valor bilateral <0,05.

**Resultados:**

À medida que o escore CAC aumentou, os níveis de sTWEAK diminuíram de forma estatisticamente significativa e detectou-se uma relação forte entre níveis de sTWEAK e escore CAC (r: -0,779, p<0,001). A análise ROC revelou que o nível de corte ideal de sTWEAK para prever o escore CAC de 400 era 761 pg/mL com uma sensibilidade de 71% e especificidade de 73% (AUC: 0,78; IC 95%: 0,70-0,85; p <0,001).

**Conclusões:**

Embora os estudos em larga escala tenham demonstrado uma correlação positiva entre os níveis de TFGe e sTWEAK, alguns estudos detectaram que o aumento nos níveis de sTWEAK estão associados a mortalidade e gravidade do sistema da artéria coronária em pacientes com DRC. Nossos resultados comprovam nossa hipótese de que os níveis de sTWEAK mostram calcificação coronária em vez de outros tipos de placas ateroscleróticas.

## Introdução

A associação entre aterosclerose e doença renal crônica (DRC) é bem estabelecida, e pacientes com DRC estão associados a um índice de mortalidade relacionada a aterosclerose 8 vezes mais alto do que o da população geral.^1 , 2^ A fisiopatologia da aterosclerose inclui anormalidades lipídicas, disfunção endotelial, envelhecimento e inflamação.^3^ O papel da inflamação e da imunidade na fisiopatologia da aterosclerose foi demonstrado nas últimas décadas.^3 - 5^ O receptor fraco indutor de apoptose semelhante a fator de necrose tumoral solúvel (sTWEAK) é um membro da superfamília de TNF que tem um papel crítico na proliferação e na inflamação.^6 - 8^ O sTWEAK foi estudado em pacientes com DRC e já se demonstrou que seu nível diminui à medida que a taxa de filtração glomerular estimada (TFGe) diminui.^9 , 10^ Embora o nível reduzido de sTWEAK tenha sido encontrado na aterosclerose, outro estudo detectou o aumento do nível de sTWEAK com a gravidade das artérias coronárias.^11^

Na DRC, o metabolismo anormal de minerais e ossos resulta no acúmulo de calcificação arterial.^12^ Devido aos resultados controversos, neste estudo prospectivo, temos o objetivo de demonstrar a relação entre o nível de sTWEAK e a calcificação da artéria coronária (CAC) em pacientes com DRC em tratamento conservador.

## Métodos

### Participantes do estudo

**Figura 1:**
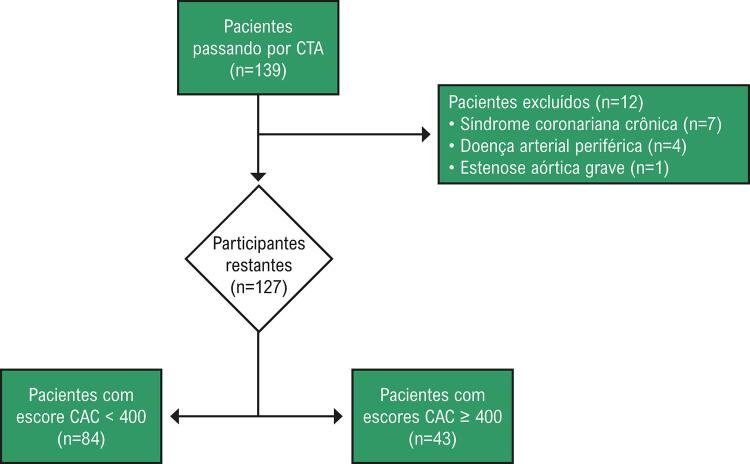
Fluxograma ilustrando a exclusão de participantes para a amostra final do estudo.

Este estudo prospectivo incluiu 139 pacientes consecutivos passando por angiografia coronariana por tomografia computadorizada (ACTC), por qualquer motivo, de agosto de 2020 a fevereiro de 2021. Todos os pacientes cadastrados neste estudo foram diagnosticados com DRC, e tinham uma taxa de filtração glomerular estimada (TFGe) abaixo de 60 por ≥3 meses ou uma TFGe acima de 60 com albuminuria (razão creatinina/albumina na urina ≥30 mg/g).^13^ A TFGe foi calculada usando-se a fórmula do estudo Modificação da dieta na doença renal (MDRD).^14^ Um total de 57 pacientes (41%) tinha DRC categoria 2; 45 (32%), DRC categoria 3a; 33 (24%), DRC categoria 3b; e 4 (0,2%), categoria 4. A população estudada não tinha histórico de aterosclerose (doença arterial coronariana, acidente vascular isquêmico, doença arterial periférica, e aneurisma torácico/abdominal). Os critérios de exclusão incluíam: (i) qualquer doença cardiovascular anterior, (ii) transplante de órgão anterior, (iii) presença de mais de uma doença valvar leve, (iv) presença de insuficiência cardíaca ou sistólica, (v) presença de disfunção diastólica diferente da disfunção diastólica grau 1, e hipertrofia do ventrículo esquerdo, (vi) presença de estenose da artéria coronária epicárdica, (vii) pacientes em hemodiálise, (viii) pacientes com síndromes coronarianas agudas. Um total de 12 pacientes foi excluído do estudo antes da ACTC por apresentarem doença arterial periférica (n=4), estenose aórtica grave (n=1), e por fazerem uso de medicamento para a síndrome coronariana crônica (n=7) ( Figura 1 ). Um total de 127 pacientes foi dividido em dois grupos com base em terem um escore CAC menor que 400 (n=84) ou um escore de 400 ou mais (n=43). Este estudo foi aprovado pelo Comitê de ética em estudos clínicos (Nº 2021/0005). O termo de consentimento informado foi obtido de todos os pacientes cadastrados neste estudo.

### Dados clínicos e demográficos

Todos os pacientes preencheram os questionários de histórico de saúde e de medicamentos, incluindo o histórico clínico de doença arterial coronariana (DAC), doença arterial periférica (DAP), diabetes mellitus (DM), hipertensão (HTN), e uso de medicamentos. Antes da CTA, todos os pacientes passaram por ecocardiograma transtorácico, ultrassom duplex da carótida, e ultrassom Doppler arterial de membro inferior para excluir a aterosclerose subclínica. Foi realizado um ecocardiograma usando-se o sistema Vivid 7 (GE Vingmed Ultrasound AS, Horten, Noruega), e a fração de ejeção ventricular esquerda (FEVE) foi calculada usando-se o método de Simpson modificado.^15^

Os valores do hemograma foram obtidos de amostras de sangue venoso coletadas na internação. O hemograma completo foi realizado usando-se um Coulter LH 780 Hematology Analyzer (Beckman Coulter Ireland, Inc., Galway, Irlanda). As medições bioquímicas foram realizadas usando-se kits e calibradores da Siemens Healthcare Diagnostic Products (Marburg, Alemanha). As amostras sanguíneas para níveis de sTWEAK plasmático foram obtidas antes da CTA e foram determinados usando-se kits ELISA (Bender MedSystems, Viena, Áustria).

### Definições

O escore Agatston é um dos sistemas de pontuação usados mais frequentemente para avaliar a calcificação da artéria coronária. Em geral, o score CAC é dividido em cinco grupos da seguinte maneira; 0, sem calcificação coronária; 1 a 100, calcificação coronária leve; >100 a 399, calcificação coronária moderada; 400 a 999, calcificação coronária grave; e ≥1.000, calcificação coronária extensa.^16 , 17^ Dividimos a população do estudo em dois grupos de pacientes com CAC de grave a extensa (n=43) e pacientes sem CAC ou com CAC leve a moderada (n=84).

### Análises estatísticas

Todos os testes estatísticos foram realizados usando-se o Statistical Package for the Social Sciences 19.0 para Windows (SPSS Inc., Chicago, IL, EUA). O teste Kolmogorov-Smirnov foi usado para analisar a normalidade dos dados. Variáveis contínuas com distribuição normal foram descritas usando-se médias ± desvio padrão (DP) e variáveis contínuas sem distribuição normal foram descritas usando-se mediana e faixa interquartil. Dados categóricos são expressos como frequência (%). Diferenças nas variáveis categóricas entre os grupos foram avaliadas com o teste qui-quadrado. As relações entre parâmetros sem distribuição normal foram avaliadas usando-se a análise de correlação de Spearman. O teste t de Student ou o teste U de Mann Whitney foram usados para comparar amostras não pareadas conforme necessário. Análises de regressão logística univariadas e multivariadas foram usadas para identificar variáveis independentes de DAC e CAC. Após realizar a análise univariada, variáveis significativas obtidas foram selecionadas nas análises de regressão logística multivariadas com o método stepwise. Os resultados das análises de regressão univariada e multivariada foram apresentados como razão de chance com 95%. Para o parâmetro laboratorial do sTWEAK, foram obtidas curvas de característica de operação do receptor (ROC), e foram selecionados os valores ideais com a sensibilidade e a especificidade mais altas na previsão do escore de cálcio coronário (400). A significância foi presumida em p-valor bilateral <0,05.

## Resultados

Um total de 127 pacientes (média de idade 59,9± 9,4 anos; homens 39%) passando por CTA cadastrados no estudo, e as características de linha de base, bem como os parâmetros laboratoriais são apresentados na Tabela 1 . Os pacientes cadastrados no estudo foram diagnosticados com DRC em estágios 3-5, e os níveis médios de TFGe, creatinina, e nitrogênio ureico sanguíneo foram de 39,9±13,1 mL/dk/1,73 m^2^, 1,8±0,2 mg/dL, e 43,5±8,4 mg/dL, respectivamente. O escore CAC por Agatston médio foi de 90 (0-1605), e 43 pacientes apresentaram um escore de >400, o que representa CAC grave a extensa ( Tabela 1 ).

**Tabela 1:** Características clínicas e laboratoriais de pacientes com doença renal crônica

	n=127
Idade, anos	59,9 ± 9,4
Sexo (masculino, %)	49 (39%)
IMC kg/m^2^	29,0 ± 3,7
HTN, n(%)	87 (68%)
DM, n(%)	53 (42%)
Tabagismo, n(%)	35 (27%)
Pressão arterial sistólica, mmHg	134,2±22,7
Pressão arterial diastólica, mmHg	77,4±11,8
Glicemia em jejum, mg/dL	119,4±47,1
Hemoglobina, g/dL	13,8±1,6
PLT, células/µL	237,7±65,2
WBC, células/µL	7,5±1,8
Creatinina, mg/dL	1,8±0,2
TFGe, ml/dk/1,73 m^2^	39,9±13,1
BUN, mg/dL	43,5±8,4
Ácido úrico, mg/dL	7,4±1,2
Sódio, mmol/L	139,6±2,3
Potássio, mmol/L	4,3±0,4
Cálcio, mmol/L	9,4±0,4
AST, U/L	22,4±9,3
ALT, U/L	23,8±11,2
PCR, mg/dL	0,3 (0,1-9,2)
Albumina, g/dL	4,3±0,4
CT, mg/dL	207,3±45,8
LDL, mg/dL	126,7±42,5
HDL, mg/dL	48,7±12,0
Tg, (mg/dL)	161,3±77,5
sTWEAK, pg/mL	845,0±418,0
Escore CAC	90 (0-1605)
Escore CAC <400, n (%)	84 (66%)
Escore CAC ≥400, n (%)	43 (34%)

*AST: aspartato aminotransferase; ALT: alanina aminotransferase; IMC: índice de massa corporal; CAC: calcificação da artéria coronária; PCR: proteína C reativa; DM: diabetes mellitus; TFGe: taxa de filtração glomerular estimada; HDL: lipoproteína de alta densidade; HTN: hipertensão; LDL: lipoproteína de baixa densidade; PLT: plaqueta; sTWEAK: receptor fraco indutor de apoptose semelhante a fator de necrose tumoral solúvel; CT: colesterol total; TG: triglicérides; WBC: leucócito. ^a^Variáveis contínuas são apresentadas como média (DP); variáveis categóricas são apresentadas como frequência (%).*

**Figura 2:**
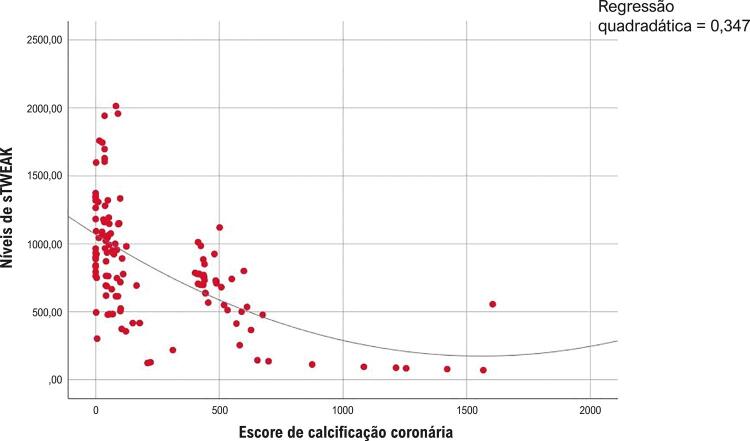
Análise de correlação do sTWEAK e escore de calcificação da artéria coronária (r:-0,615, p<0,001, boa correlação).

A relação entre níveis de sTWEAK e o escore CAC foi avaliada pela análise de correlação de Spearman. À medida que o escore CAC aumentou, os níveis de sTWEAK diminuíram significativamente e detectou-se uma relação forte entre níveis de sTWEAK e escore CAC, que é mostrada na Figura 2 (r: -0,615, p<0,001).

Os participantes foram divididos em dois grupos, pacientes com um escore CAC <400 (n=84) pacientes com um escore CAC ≥400 (n=43). Não houve diferenças estatisticamente significativas entre os grupos em relação a idade, sexo, IMC, HTN, DM, ou tabagismo, conforme mostrado na Tabela 2 . Os parâmetros laboratoriais, tais como glicemia em jejum, Hgb, WBC de plaqueta, creatinina, TFGe, ácido úrico, sódio, potássio, CT, LDL, HDL, Tg, não representaram diferenças estatisticamente significativas ( Tabela 2 ).

**Tabela 2:** Características clínicas e laboratoriais dos pacientes divididos em dois grupos com base no escore de calcificação da artéria coronáriaa

	SCC≥400 (n=43)	SCC <400 (n=84)	p
Idade, anos	61,7 ± 8,8	59,0 ± 9,6	0,126
Sexo (masculino%)	19(44%)	30(35%)	0,353
IMC kg/m^2^	29,8 ± 3,8	28,6± 3,6	0,098
HT, n(%)	33(78%)	54(65%)	0,121
DM, n(%)	22(52%)	31(37%)	0,108
Tabagismo, n(%)	15(36%)	20(25%)	0,198
Pressão arterial sistólica, mmHg	137,5±20,7	132,4±23,6	0,241
Pressão arterial diastólica, mmHg	80,0±11,5	76,0±11,7	0,073
Glicemia em jejum, mg/dL	122,8±62,9	117,5±24,5	0,601
Hemoglobina, g/dL	13,6±1,4	13,9±1,7	0,336
PLT, células/µL	235,5±68,1	239,0±63,9	0,797
WBC, cédulas/µL	7,4±1,8	7,5±1,9	0,687
Creatinina, mg/dL	1,8±0,2	1,9±0,1	0,887
Estágios da DRC			
Estágio 2	18	36	0,914
Estágio 3a	15	27	0,756
Estágio 3b	10	18	0,814
Estágio 4	1	2	0,984
TFGe, ml/dk/1,73 m^2^	39,8±13,6	40,2±12,9	0,890
BUN, mg/dL	36,4±9,3	32,0±7,4	0,009
Ácido úrico, mg/dL	7,5±1,1	7,3±1,3	0,274
Sódio, mmol/L	139,5±2,2	139,6±2,3	0,825
Potássio, mmol/L	4,2±0,5	4,3±0,3	0,115
Cálcio, mmol/L	9,3±0,3	9,4±0,4	0,066
AST, U/L	22,2±8,1	22,6±11,3	0,875
ALT, U/L	23,5±11,1	24,0±10,0	0,898
PCR, mg/dL	0,3 (0,1-9,2)	0,2 (0,1-2,0)	0,009
Albumina, g/dL	4,1±0,5	4,4±0,2	0,005
CT, mg/dL	215,8±41,6	202,9±47,5	0,132
LDL, mg/dL	135,2±40,0	122,4±43,4	0,108
HDL, mg/dL	49,6±13,3	48,3±11,3	0,550
Tg, (mg/dL)	158,4±59,1	162,8±85,7	0,758
sTWEAK, pg/mL	586,2±286,6	977,5±413,8	<0,001
Escore CAC	488 (402-1605)	45 (0-312)	<0,001

*AST: aspartato aminotransferase; ALT: alanina aminotransferase; IMC: índice de massa corporal; CAC: calcificação da artéria coronária; PCR: proteína C reativa; DM: diabetes mellitus; TFGe: taxa de filtração glomerular estimada; HDL: lipoproteína de alta densidade; HTN: hipertensão; LDL: lipoproteína de baixa densidade; PLT: plaqueta; CT: colesterol total; Tg: triglicérides; sTWEAK: receptor fraco indutor de apoptose semelhante a fator de necrose tumoral solúvel; WBC: leucócito. ^a^Variáveis contínuas são apresentadas como média (DP); variáveis categóricas são apresentadas como frequência (%).*

**Figura 3:**
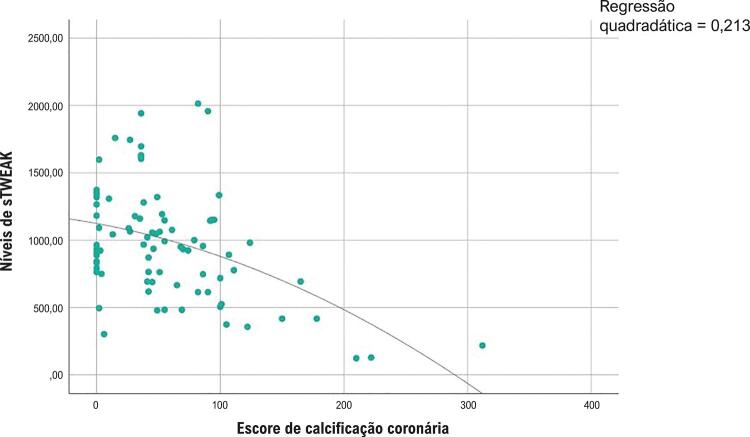
Análise de correlação do sTWEAK e escore de calcificação da artéria coronária de menos de 400 (r:-0,385, p<0,001, correlação moderada).

**Figura 4:**
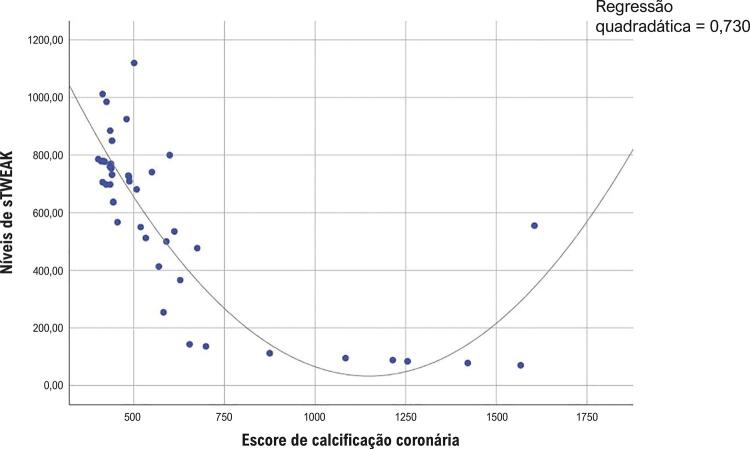
Análise de correlação do sTWEAK e escore de calcificação da artéria coronária de 400 ou mais (r:-0,779, p<0,001, correlação forte).

**Figura 5:**
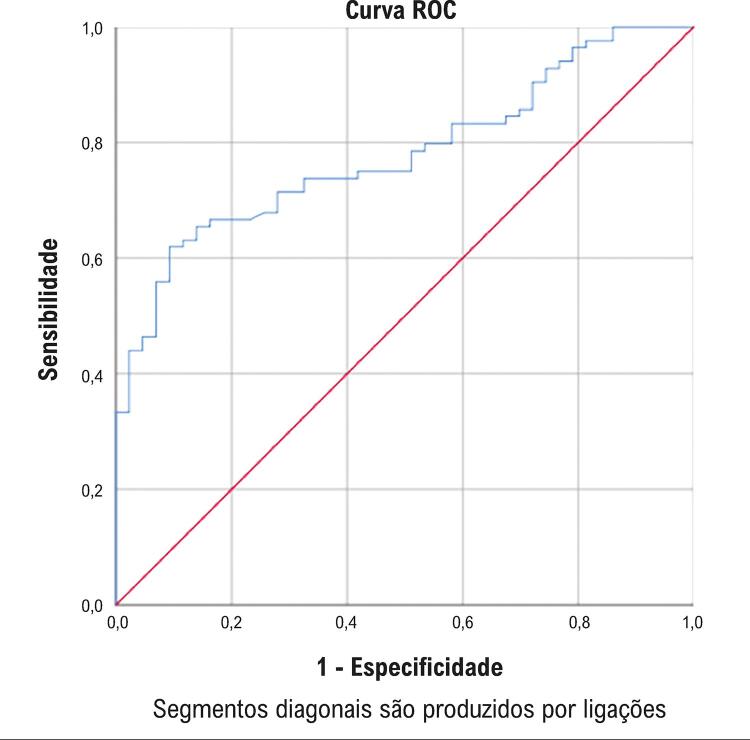
A análise ROC revelou que o valor de corte ideal de sTWEAK para prever o escore CAC de ≥ 400 era 761 pg/mL com uma sensibilidade de 71% e especificidade de 73% (AUC= 0,78; IC 95%: 0,70-0,85; p <0,001).

O nível de sTWEAK foi significativamente menor no grupo com um escore CAC ≥400 que no grupo com um escore CAC <400 ( Tabela 2 ). A relação entre níveis de sTWEAK e o escore CAC em pacientes com escores CAD mais baixos (<400) foi avaliada pela análise de correlação de Spearman. À medida que o escore CAC aumentou, os níveis de sTWEAK diminuíram de maneira estatisticamente significativa, e detectou-se uma relação moderada entre níveis de sTWEAK e escore CAC, que é mostrada na Figura 3 (r: -0,385, p<0,001). A relação entre níveis de sTWEAK e o escore CAC em pacientes com escores CAD mais altos (≥400) foi avaliada pela análise de correlação de Spearman. À medida que o escore CAC aumentou, os níveis de sTWEAK diminuíram de maneira estatisticamente significativa, e detectou-se uma relação forte entre níveis de sTWEAK e escore CAC, que é mostrada na Figura 4 (r: -0,779, p<0,001). Avaliamos a especificidade e a sensibilidade dos níveis de sTWEAK pela análise da característica de operação do receptor (ROC) para prever a presença do escore CAC de 400. A análise ROC revelou que o nível de corte ideal de sTWEAK para prever o escore CAC de 400 era 761 pg/mL com uma sensibilidade de 71% e especificidade de 73% (AUC: 0,78; IC 95%: 0,70-0,85; p <0,001) ( Figura 5 ).

Os parâmetros que afetam o desenvolvimento da CAC foram avaliados por análise univariada e multivariada. Os prováveis preditores de DAC, tais como idade, sexo, HTN, DM, DRC, tabagismo, IMC, PCR, LDL, e sTWEAK foram avaliados na análise univariada. Na análise multivariada, idade, tabagismo, LDL e sTWEAK foram associados ao escore CAC de 400 (RC da idade: 1,033, p: 0,003; RC tabagismo: 4,638, p: 0,003; RC LDL: 1,016, p: 0,005; RC sTWEAK: 0,345, p<0,001) ( Tabela 3 ).

**Tabela 3:** Preditores univariados e modelo multivariado para o escore de calcificação da artéria coronária de 400

Variável	Univariada			Multivariada		

	RC	IC95%	p	RC	IC95%	p
Idade	1,049	1,009-1,091	0,016	1,033	1,016-1,058	0,003
Sexo	0,567	0,276-1,166	0,123			
HTN	1,093	0,492-2,265	0,877			
DM	0,591	0,289-1,209	0,150			
DRC	1,105	0,151-8,105	0,922			
Tabagismo	4,552	1,898-10,915	0,001	4,638	1,965-11,236	0,003
IMC	0,969	0,881-1,066	0,513			
PCR	2,490	0,802-7,731	0,314			
LDL	1,017	1,007-1,027	0,001	1,016	1,005-1,028	0,005
sTWEAK	0,314	0,172-0,507	<0,001	0,345	0,201-0,581	<0,001

*IMC: índice de massa corporal; DRC: doença renal crônica; PCR: proteína C reativa; DM: diabetes mellitus; HTN: hipertensão; LDL: lipoproteína de baixa densidade; sTWEAK: receptor fraco indutor de apoptose semelhante a fator de necrose tumoral solúvel.*

## Discussão

Os pacientes com o escore CAC de 400 têm um risco alto de eventos cardíacos adversos (>2% por ano), e um terço desses pacientes têm imagens de perfusão miocárdica anormais.^18 , 19^ Neste estudo, a população do estudo foi dividida em dois grupos com base em seu escore CAC. À medida que o escore CAC aumentou, o sTWEAK diminuiu de forma estatisticamente significativa, especialmente em pacientes com um escore de 400 (r: -779, p<0,001, correlação forte) ( Figura 2-4 ). Os níveis de sTWEAK mais baixos continuaram a ser um preditor de escore alto de CAC na análise multivariada ( Tabela 2 ).

A placa aterosclerótica consiste em mediadores pró-inflamatórios, citocinas e quimiocinas.^20 , 21^ As citocinas podem desestabilizar a placa e aumentar o risco de eventos trombóticos.^22 - 24^ O sTWEAK é um dos mensageiros inflamatórios que contribui para a formação da placa aterosclerótica, e detectou-se que o nível alto de sTWEAK está associado à gravidade das artérias coronárias em pacientes com síndrome coronariana crônica.^11^ Vários estudos em animais corroboraram esses achados que demonstraram a relação entre o sTWEAK e as atividades pró-trombóticas.^6 , 7 , 25^ Além disso, identificou-se que o tratamento anti-TWEAK reduz o avanço das placas ateroscleróticas e a inflamação em modelos animais.^6 , 25^

A relação inversa foi demonstrada na aterosclerose nas artérias carótidas em pacientes em hemodiálise.^26^ A associação também foi encontrada na aterosclerose carótida em pacientes com infecção por HIV.^27^ Em vários estudos, a redução gradual no nível de sTWEAK foi observada com a redução da TFGe.^9 , 28 , 29^ Embora tenha-se levantado a hipótese de que o nível aumentado de sTWEAK pudesse refletir vasos saudáveis, detectou-se que o nível de sTWEAK aumentado em pacientes em hemodiálise é um preditor de mortalidade.^30^ Ainda há uma controvérsia sobre se o nível alto ou baixo de sTWEAK está associado a aterosclerose. Vários estudos demonstraram que o nível de sTWEAK era mais baixo em pacientes com DRC com aterosclerose e observaram uma diminuição contínua no nível de sTWEAK após o acompanhamento por 2 anos.^10 , 28 , 31^ Resultados opostos foram identificados em outro estudo, que demonstrou que um aumento no nível de sTWEAK estava associado a um escore de Gensini alto.^11^

Placas ateroscleróticas geralmente desenvolvem calcificações. Sabe-se que membros da família TNF, tais como o ligante do receptor ativador de NF-B (RANKL), promovem a formação de cálcio em placas ateroscleróticas.^3^ Pacientes com DRC têm placas coronárias calcificadas mais graves do que aqueles sem DRC.^31 - 33^ À medida que a TFGe diminui, especialmente para menos de 60 mL/min/1,73 m^2^, a capacidade da eliminação das quedas de fósforo. Ele acaba reduzindo os níveis de 1,25-dihidroxicholecalciferol, que causa hipocalcemia relativa. Essa hipocalcemia pode desencadear a liberação do hormônio paratormônio, causando o acúmulo de cálcio no sistema vascular.^12^ Sastre C et al.^6^ identificaram que o sTWEAK pode diminuir a carga da calcificação da placa, e isso pode explicar a inconsistência dos estudos em termos dos níveis de sTWEAK em pacientes com aterosclerose. O estudo encontrou uma correlação positiva com a gravidade das artérias coronárias, incluindo pacientes com DRC de leves a moderadas, e os pesquisadores avaliaram angiografias coronárias invasivas convencionais.^11^ Eles não utilizaram a CTA, que é uma ferramenta excelente para mostrar calcificações das artérias coronárias. Neste estudo, analisamos um grupo homogêneo de pacientes com DRC com e sem calcificações coronárias. Nossos resultados comprovam nossa hipótese de que os níveis de sTWEAK mostram calcificação coronária em vez de aterosclerose.

### Limitações

Este estudo tem possíveis limitações. Primeiramente, nossa população se limitava a pacientes com DRC. Portanto, nossos resultados não podem ser generalizados para todos os pacientes com aterosclerose. Em segundo lugar, o número de pacientes do estudo era relativamente pequeno, e, portanto, estudos posteriores em grande escala são necessários para confirmar esses achados. Terceiro, o estudo foi realizado em um único hospital terciário universitário. Portanto, havia a possibilidade de viés de seleção, embora tenha-se prestado muita atenção para garantir que todos os pacientes consecutivos que estavam passando por CTA fossem incluídos para evitar tal viés de seleção. Além disso, o viés interobservador pode ser alto no escore Agatston, que foi usado para calcular a carga de calcificação.

## Conclusões

Embora os estudos em larga escala tenham demonstrado uma correlação positiva entre os níveis de TFGe e sTWEAK, alguns estudos detectaram que o aumento nos níveis de sTWEAK estão associados a mortalidade e gravidade do sistema da artéria coronária em pacientes com DRC. Nossos resultados comprovam nossa hipótese de que os níveis de sTWEAK mostram calcificação coronária em vez de outros tipos de placas ateroscleróticas.
