# Supplementary material for: A Associação de TWEAK com Calcificação da Artéria Coronária em Pacientes com Doença Renal Crônica
Source: Arq Bras Cardiol. 2022 Jun 6;119(3):436–45. [Article in Portuguese] doi: 10.36660/abc.20210599 (PMC9438529; doi:10.36660/abc.20210599)
Supplement: Translation

# Translation: The Association of TWEAK with Coronary Artery Calcification in Patients with Chronic Kidney Disease

**Authors:** Mustafa Adem Tatlisu, Adem Atici, Fatma Betul Ozcan, Mehmet Çelik, Eray Kirac, Omer Faruk Baycan, Mustafa Caliskan

**Affiliations:** 1 Turkey Istanbul Medeniyet University, Istanbul – Turkey

**Keywords:** Cardiovascular Diseases, Renal Insufficiency,Chronic, Vascular Stiffness, Atherosclerosis, Coronary Artery Disease

## Abstract

**Background:**

The soluble tumor necrosis factor-like weak inducer of apoptosis (sTWEAK) is a member of the TNF superfamily that plays a critical role in proliferation and inflammation in the arterial circulation.

**Objectives:**

This prospective study aimed to show the relationship between the sTWEAK levels and coronary artery calcification (CAC) in patients with chronic kidney disease (CKD).

**Methods:**

This prospective study included 139 consecutive patients undergoing computed coronary angiography for any reason except for acute coronary syndromes from August 2020 to February 2021. A total of 12 patients were excluded from the study due to exclusion criteria. Patients were divided into two groups with regard to having a CAC score of less than 400 (n=84) and 400 or more (n=43). Significance was assumed at a 2-sided p<0.05.

**Results:**

As the CAC score increased, sTWEAK levels presented a statistically significant decrease, and a strong relationship between sTWEAK levels and the CAC score (r: -0.779, p<0.001) was observed. The ROC analysis revealed that the optimal cut-off level of sTWEAK for predicting the CAC score of 400 was 761 pg/mL with a sensitivity of 71% and a specificity of 73% (AUC: 0.78; 95% CI:0.70-0.85; p < 0.001)

**Conclusions:**

Even though the large-scale studies showed a positive correlation between eGFR and the sTWEAK levels, some studies found the increased sTWEAK levels to be associated with mortality and the severity of the coronary artery system in patients with CKD. Our results support our hypothesis that the sTWEAK level shows coronary calcification rather than other types of atherosclerotic plaques.

## Introductıon

The association of atherosclerosis with chronic kidney disease (CKD) is well-established, and the patients with CKD are associated with a more than 8-fold atherosclerosis-related death rate than in the general population.^1 , 2^ The pathophysiology of atherosclerosis includes lipid abnormalities, endothelial dysfunction, aging, and inflammation.^3^ The role of inflammation and immunity in the pathophysiology of atherosclerosis has been demonstrated in recent decades.^3 - 5^ The soluble tumor necrosis factor-like weak inducer of apoptosis (sTWEAK) is a member of the TNF superfamily that plays a critical role in proliferation and inflammation.^6 - 8^ The sTWEAK has been studied in patients with CKD, and it has been shown that its’ level declines as the estimated glomerular filtration rate (eGFR) decreases.^9 , 10^ Even though the decreased sTWEAK level was found in atherosclerosis, another study found the association of increased sTWEAK level with severity of coronary arteries.^11^

In CKD, the abnormal metabolism of minerals and bones results in the accumulation of arterial calcification.^12^ On account of the controversial results, this prospective study aimed to show the relationship between the sTWEAK level and coronary artery calcification (CAC) in patients with CKD under conservative treatment.

## Methods

### Study participants

This prospective study included 139 consecutive patients undergoing computed coronary angiography (CCA) for any reason from August 2020 to February 2021. All patients enrolled in the study were diagnosed with CKD, who had an estimated glomerular filtration rate (eGFR) below 60 for ≥ 3 months or an eGFR above 60 with albuminuria (urine albumin/creatinine ratio ≥ 30 mg/g).^13^ The eGFR was calculated using the Modification of Diet in Renal Disease (MDRD) formula.^14^ A total of 57 patients (41%) had category 2 CKD; 45 (32%), category 3a CKD; 33 (24%), category 3b CKD; and 4 (0.2%), category 4. The studied population had no history of atherosclerosis (coronary artery disease, ischemic stroke, peripheral artery disease, and thoracic/abdominal aneurysm). The exclusion criteria included: (i) any previous cardiovascular disease, (ii) previous organ transplantation, (iii) presence of more than a mild valvular disease, (iv) presence of systolic or heart failure, (v) presence of diastolic dysfunction other than grade 1 diastolic dysfunction and left ventricular hypertrophy, (vi) presence of epicardial coronary artery stenosis, (vii) patients on hemodialysis, and (viii) patients with acute coronary syndromes. A total of 12 patients were excluded from the study before the CCA, as they presented peripheral artery disease (n=4), showed severe aortic stenosis (n=1), and were taking medication for the chronic coronary syndrome (n=7) ( Figure 1 ). A total of 127 patients were divided into two groups based on having coronary artery calcium (CAC) scores of less than 400 (n=84) and 400 or more (n=43). This study was approved by the local Clinical Studies Ethics Committee (No: 2021/0005). Informed consent was obtained from all patients enrolled in this study.

**Figure 1:** The flowchart illustrating the exclusion of participants for the final study sample.

### Demographic and clinical data

All patients completed the health and medication history questionnaires, including the clinical history of coronary artery disease (CAD), peripheral artery disease (PAD), Diabetes Mellitus (DM), hypertension (HTN), and medication use. Before CTA, all patients underwent

transthoracic echocardiography, carotid duplex ultrasound, and lower extremity arterial Doppler ultrasound to exclude subclinical atherosclerosis. An echocardiogram was performed using a Vivid 7 system (GE Vingmed Ultrasound AS, Horten, Norway), and left ventricular ejection fraction (LVEF) was calculated using the modified Simpson method.^15^

Blood values were obtained from venous blood samples upon hospital admission. The complete blood count was measured by using a Coulter LH 780 Hematology Analyzer (Beckman Coulter Ireland, Inc., Galway, Ireland). Biochemical measurements were performed by using Siemens Healthcare Diagnostic Products kits and calibrators (Marburg, Germany). The blood samples for plasma sTWEAK levels were obtained before the CTA and were determined using ELISA kits (Bender MedSystems, Vienna, Austria).

### Definitions

The Agatston score is one of the most frequently used scoring systems to assess coronary artery calcification. In general, the CAC score is divided into five groups as: 0, no coronary calcification; 1-100, mild coronary calcification; >100 to 399, moderate calcification; 400 to 999, severe calcification; and ≥1,000, extensive calcification.^16 , 17^ We divided the study population into two groups as patients with severe to extensive CAC (n=43) and patients without any CAC or mild to moderate CAC (n=84).

### Statistical analyses

All statistical tests were conducted using the Statistical Package for the Social Sciences 19.0 for Windows (SPSS Inc., Chicago, IL, USA). The Kolmogorov-Smirnov test was used to analyze the normality of the data. Continuous variables with normal distribution were described using mean ± standard deviation (SD); and continuous variables without normal distribution were described using median and interquartile range. The categorical data are expressed as frequency (%). The Chi-square test was used to assess differences in categorical variables between groups. The relationships among parameters without normal distribution were assessed using Spearman’s correlation analysis. The Student’s t-test or Mann Whitney U test was used to compare unpaired samples as needed. Univariate and multivariate logistic regression analysis were used to identify independent variables of CAD and CAC. After performing univariate analysis, significantly obtained variables were selected into the multivariate logistic regression analyses with the stepwise method. The results of univariate and multivariate regression analyses were presented as odds ratio with 95%. For the laboratory parameter of sTWEAK receiver operating characteristic (ROC) curves were obtained, and the optimal values with the greatest total sensitivity and specificity in the prediction of coronary calcium score (≥400) were selected. Significance was assumed at a 2-sided p<0.05.

## Results

A total of 127 patients (mean age 59.9± 9.4 years; men 39%) undergoing CTA enrolled in the study, and the baseline characteristics and laboratory parameters are shown in Table 1 . The patients enrolled in the study were diagnosed with CKD stages 3-5, and the mean eGFR, creatinine, blood urea nitrogen levels were 39.9±13.1 mL/dk/1.73 m2, 1.8±0.2 mg/dL, 43.5±8.4 mg/dL, respectively. The mean Agatston CAC score was 90 (0-1605), and 43 patients had a score of >400, which represents severe to extensive CAC ( Table 1 ).

**Table 1:** Clinical and laboratory characteristics of patients with chronic kidney disease

	n=127
Age, years	59.9 ± 9.4
Gender (male, %)	49 (39%)
BMI, kg/m^2^	29.0 ± 3.7
HTN, n(%)	87 (68%)
DM, n(%)	53 (42%)
Smoking, n(%)	35 (27%)
Systolic blood pressure, mmHg	134.2±22.7
Diastolic blood pressure, mmHg	77.4±11.8
Fasting blood glucose, mg/dL	119.4±47.1
Hemoglobin, g/dL	13.8±1.6
PLT, cells/µL	237.7±65.2
WBC, cells/µL	7.5±1.8
Creatinine, mg/dL	1.8±0.2
eGFR, ml/dk/1.73 m^2^	39.9±13.1
BUN, mg/dL	43.5±8.4
Uric acid, mg/dL	7.4±1.2
Sodium, mmol/L	139.6±2.3
Potassium, mmol/L	4.3±0.4
Calcium, mmol/L	9.4±0.4
AST, U/L	22.4±9.3
ALT, U/L	23.8±11.2
CRP, mg/dL	0.3 (0.1-9.2)
Albumine, g/dL	4.3±0.4
TC, mg/dL	207.3±45.8
LDL, mg/dL	126.7±42.5
HDL, mg/dL	48.7±12.0
Tg, mg/dL	161.3±77.5
sTWEAK , pg/mL	845.0±418.0
CAC score	90 (0-1605)
CAC score <400, n(%)	84 (66%)
CAC score ≥400, n(%)	43 (34%)


*AST: aspartate aminotransferase; ALT: alanine aminotransferase; BMI: body mass index; CAC: coronary artery calcification; CRP: C-reactive protein; DM: diabetes mellitus; eGFR: estimated glomerular filtration rate; HDL: high-density lipoprotein; HTN: hypertension; LDL: low-density lipoprotein; Plt: platelet; TC: total cholesterol; TG: triglyceride; sTWEAK: soluble tumor necrosis factor-like weak inducer of apoptosis; WBC: white blood cell.^a^Continuous variables are presented as mean (SD); categorical variables presented as frequency (%).*

The relationship between sTWEAK levels and the CAC score was evaluated by Spearman correlation analysis. As the CAC score increased, sTWEAK levels decreased significantly, and there was a good relationship between sTWEAK levels and the CAC score, which is shown in Figure 2 (r: -0.615, p<0.001).

**Figure 2:** The correlation analysis of sTWEAK and the coronary artery calcification score (r:-0.615, p<0.001, good correlation).

The participants were divided into two groups as patients with a CAC score of <400 (n=84) and patients with a CAC score of ≥400 (n=43). There were no statistically significant differences between the groups concerning age, gender, BMI, HTN, DM, and smoking status, as shown in Table 2 . The laboratory parameters, such as fasting glucose, Hgb, platelet WBC, creatinine, eGFR, uric acid, sodium, potassium, TC, LDL, HDL, Tg, showed no statistically significant differences ( Table 2 ).

**Table 2:** Clinical and laboratory characteristics of patients divided into two groups with regard to coronary artery calcification scorea

	CCS≥400 (n=43)	CCS<400 (n=84)	p
Age, years	61.7 ± 8.8	59.0 ± 9.6	0.126
Gender (male%)	19(44%)	30(35%)	0.353
BMI, kg/m^2^	29.8 ± 3.8	28.6± 3.6	0.098
HT, n(%)	33(78%)	54(65%)	0.121
DM, n(%)	22(52%)	31(37%)	0.108
Smoking, n(%)	15(36%)	20(25%)	0.198
Systolic blood pressure, mmHg	137.5±20.7	132.4±23.6	0.241
Diastolic blood pressure, mmHg	80.0±11.5	76.0±11.7	0.073
Fasting blood glucose, mg/dL	122.8±62.9	117.5±24.5	0.601
Hemoglobin, g/dL	13.6±1.4	13.9±1.7	0.336
PLT, cells/µL	235.5±68.1	239.0±63.9	0.797
WBC, cells/µL	7.4±1.8	7.5±1.9	0.687
Creatinine, mg/dL	1.8±0.2	1.9±0.1	0.887
Stages of CKD			
Stage 2	18	36	0.914
Stage 3a	15	27	0.756
Stage 3b	10	18	0.814
Stage 4	1	2	0.984
eGFR, ml/dk/1.73 m^2^	39.8±13.6	40.2±12.9	0.890
BUN, mg/dL	36.4±9.3	32.0±7.4	0.009
Uric acid, mg/dL	7.5±1.1	7.3±1.3	0.274
Sodium, mmol/L	139.5±2.2	139.6±2.3	0.825
Potassium, mmol/L	4.2±0.5	4.3±0.3	0.115
Calcium, mmol/L	9.3±0.3	9.4±0.4	0.066
AST, U/L	22.2±8.1	22.6±11.3	0.875
ALT, U/L	23.5±11.1	24.0±10.0	0.898
CRP, mg/dL	0.3 (0.1-9.2)	0.2 (0.1-2.0)	0.009
Albumine, g/dL	4.1±0.5	4.4±0.2	0.005
TC, mg/dL	215.8±41.6	202.9±47.5	0.132
LDL, mg/dL	135.2±40.0	122.4±43.4	0.108
HDL, mg/dL	49.6±13.3	48.3±11.3	0.550
Tg, mg/dL	158.4±59.1	162.8±85.7	0.758
sTWEAK, pg/mL	586.2±286.6	977.5±413.8	<0.001
CAC score	488 (402-1605)	45 (0-312)	<0.001

*AST: aspartate aminotransferase; ALT: alanine aminotransferase; BMI: body mass index; CAC: coronary artery calcification; CRP: C-reactive protein; DM: diabetes mellitus; eGFR: estimated glomerular filtration rate; HDL: high-density lipoprotein; HTN: hypertension; LDL: low-density lipoprotein; PLT: platelet; sTWEAK: soluble tumor necrosis factor-like weak inducer of apoptosis; TC: total cholesterol; TG: triglyceride; WBC: white blood cell. aContinuous variables are presented as mean (SD); categorical variables presented as frequency (%).*

The sTWEAK level was significantly lower in the group with a CAC score of ≥400 than the group with a CAC score of <400 ( Table 2 ). The relationship between sTWEAK levels and the CAC score in patients with lower CAC scores (<400) was evaluated by Spearman correlation analysis. As the CAC score increased, sTWEAK levels decreased significantly, and there was a moderate relationship between sTWEAK levels and the CAC score, which is shown in Figure 3 (r: -0.385, p<0.001). The relationship between sTWEAK levels and the CAC score in patients with higher CAC scores (≥400) was evaluated by Spearman correlation analysis. As the CAC score increased, sTWEAK levels decreased significantly, and there was a strong relationship between sTWEAK levels and the CAC score, which is shown in Figure 4 (r: -0.779, p<0.001). We evaluated the specificity and sensitivity of the sTWEAK levels by Receiver Operating Characteristic (ROC) analysis to predict the presence of the CAC score of 400. The ROC analysis revealed that the optimal cut-off level of sTWEAK for predicting the CAC score of 400 was 761 pg/mL, with a sensitivity of 71% and specificity of 73% (AUC: 0.78; 95% CI:0.70-0.85; p < 0.001) ( Figure 5 ).

**Figure 3:** The correlation analysis of sTWEAK and the coronary artery calcification score of less than 400 (r:-0.385, p<0.001, moderate correlation).

**Figure 4:** The correlation analysis of sTWEAK and the coronary artery calcification score of 400 or more (r: -0.779, p<0.001, strong correlation).

**Figure 5:** The ROC analysis revealed that the optimal cut-off value of the sTWEAK to predict the CAC score of ≥ 400 was 761 pg/mL with a sensitivity of 71% and specificity of 73% (AUC=0.78; 95% CI:0.70-0.85; p<0.001).

The parameters affecting the development of CAC were evaluated by univariate and multivariate analysis. The probable predictors of CAD, such as age, gender, HTN, DM, CKD, smoking, BMI, CRP, LDL, and sTWEAK were evaluated in the univariate analysis. In the multivariate analysis, age, smoking, LDL, and sTWEAK were associated with the CAC score of 400 (Age OR:1.033, p: 0.003; smoking OR: 4.638, p: 0.003; LDL OR:1.016, p: 0.005; sTWEAK OR: 0.345, p<0.001) ( Table 3 ).

**Table 3:** Univariate predictors and multivariate model for the coronary artery calcification score of ≥400

Variable	Univariate			Multivariate		

	OR	95%CI	p	OR	95%CI	p
Age	1.049	1.009-1.091	0.016	1.033	1.016-1.058	0.003
Gender	0.567	0.276-1.166	0.123			
HTN	1.093	0.492-2.265	0.877			
DM	0.591	0.289-1.209	0.150			
CKD	1.105	0.151-8.105	0.922			
Smoking	4.552	1.898-10.915	0.001	4.638	1.965-11.236	0.003
BMI	0.969	0.881-1.066	0.513			
CRP	2.490	0.802-7.731	0.314			
LDL	1.017	1.007-1.027	0.001	1.016	1.005-1.028	0.005
sTWEAK	0.314	0.172-0.507	<0.001	0.345	0.201-0.581	<0.001

*BMI: body mass index; CKD: chonic kidney disease; CRP: C-reactive protein; DM: diabetes mellitus; HTN: hypertension; LDL: low-density lipoprotein; sTWEAK: soluble tumor necrosis factor-like weak inducer of apoptosis.*

## Dıscussıon

The patients with the CAC score of 400 have a high risk for adverse cardiac events (>2% per year), and one-third of those patients have abnormal myocardial perfusion imaging.^18 , 19^ In our study, the study population was divided into two groups regarding their CAC scores. As the score of CAC increased, the sTWEAK decreased in a statistically significant manner, especially in patients with a score of 400 (r: -779, p<0.001, strong correlation) ( Figure 2-4 ). The lower sTWEAK levels remained an independent predictor of a high CAC score in the multivariate analysis ( Table 2 ).

The atherosclerotic plaque consists of proinflammatory mediators, cytokines, and chemokines.^20 , 21^ The cytokines can destabilize the plaque and increase the risk of thrombotic events.^22 - 24^ The sTWEAK is one of the inflammatory messengers that contributes to atherosclerotic plaque formation, and the high level of sTWEAK was found to be associated with the severity of coronary arteries in patients with the chronic coronary syndrome.^11^ Several animal studies supported these findings, which showed the relationship between the sTWEAK and prothrombic activities.^6 , 7 , 25^

Furthermore, anti-TWEAK treatment was found to reduce atherosclerotic plaque progression and inflammation in animal models.^6 , 25^

The inverse relationship was shown in atherosclerosis in carotid arteries in patients on hemodialysis.^26^ This association was also found in carotid atherosclerosis in patients with HIV infection.^27^ In several studies, the gradual reduction in the level of sTWEAK was observed as eGFR declined.^9 , 28 , 29^ Even though it was hypothesized that the increased level of sTWEAK might reflect healthy vessels, the increased sTWEAK level in patients on hemodialysis was found to be a predictor of mortality.^30^ It is still controversial whether the high or low sTWEAK level is associated with atherosclerosis. Several studies found that the sTWEAK level was lower in CKD patients with atherosclerosis and observed a continuous decrease in the sTWEAK level after a 2-year follow-up.^10 , 28 , 31^ The opposite results were found in another study, which showed that an increase in the sTWEAK level was associated with a high Gensini score.^11^

Atherosclerotic plaques usually develop calcifications. The member of the TNF family, such as the Receptor activator of NF-κB ligand (RANKL), is known to promote calcium formation in atherosclerotic plaques.^3^ The patients with CKD have more severe calcified coronary plaques than those without CKD.^31 - 33^ As the eGFR declines, especially at below 60 mL/min/1.73 m2, the capacity of elimination of phosphorus falls. It ends up reducing 1,25 dihydroxy-vitamin D levels, which causes relative hypocalcemia. This hypocalcemia can trigger the release of parathyroid hormone, causing the accumulation of calcium in the vascular system.^12^ . Sastre C et al.^6^ found that sTWEAK might decrease the burden of calcification of the plaque; this may explain the inconsistency of the studies in terms of the sTWEAK levels in patients with atherosclerosis. The study found a positive correlation with the severity of coronary arteries, including the mild to moderate CKD patients, and the investigators assessed the conventional invasive coronary angiograms.^11^ They did not use the CTA, which is excellent to show calcifications of the coronary arteries. The present study analyzed a homogenous group of CKD patients with and without coronary calcifications. Our results support that the sTWEAK level shows coronary calcification rather than atherosclerosis.

### Limitations

This study has potential limitations. First, our population was limited to patients with CKD. Hence, our results cannot be generalized to all patients with atherosclerosis. Second, the number of study patients was relatively small; therefore, further larger-scale studies are needed to confirm these findings. Third, the study was carried out in a single tertiary university hospital. Hence, there was a possibility of selection bias, although great attention was paid to include all consecutive patients undergoing CTA to avoid selection bias. Furthermore, interobserver bias could be high in the Agatston score, which was used to calculate the burden of calcification.

## Conclusions

Even though the large-scale studies showed a positive correlation between eGFR and the sTWEAK levels, some studies found the increased sTWEAK levels to be associated with mortality and the severity of the coronary artery system in patients with CKD. Our results support our hypothesis that the sTWEAK level shows coronary calcification rather than other types of atherosclerotic plaques.
